# Recognising dog movement with behaviour-specific machine learning models: bout length as a biologically relevant parameter for window size

**DOI:** 10.1186/s12917-026-05294-1

**Published:** 2026-03-19

**Authors:** Gábor Csizmadia, Bálint Daróczy, Bence Ferdinandy, Balázs Nagy, Ádám Miklósi

**Affiliations:** 1 Department of Ethology, Eötvös Lóránd University, Budapest, Hungary; 2 AI Laboratory, Institute for Computer Science and Control, HUN-REN SZTAKI, Budapest, Hungary; 3 Department of Mathematical Engineering, Université catholique de Louvain, INMA, Louvain-La-Neuve, Belgium; 4 MTA-ELTE Comparative Ethology Research Group, Budapest, Hungary; 5 Department of Artificial Intelligence, Eötvös Lóránd University, Budapest, Hungary

**Keywords:** Machine learning, Supervised learning, One-vs-All, LGBM, Window size, Bout length, Behaviour recognition

## Abstract

**Background:**

Machine learning methods are widely used to detect behavioural data patterns. Although these new mathematical methods are useful tools, the interpretation of the results are often ambivalent unless biologically relevant parameters are included in the analyses. In case of classical (non-neural) machine learning (ML) methods, a crucial first step in time series data analysis is to determine the window length for which the features are computed as input variables for the ML training phase. The bout length of behaviours could be a relevant parameter to determine the window length used by the machine learning methods.

**Methods:**

In this research the movements of dogs were observed. Eight behaviours were defined and motion data was collected using a smartwatch attached to the collar of the dogs. The behaviour sequences of 56 freely moving dogs of various breeds were analysed by using a specific software (SensDog by CEM Inc.). Behaviour recognition was based on binary classification evaluated with a Light Gradient Boosted Machine (LGBM) learning algorithm. For signal processing, sliding window technique was used to find the best window size for the analysis of each behavior.

**Results:**

Results showed that for all behaviours, the best recognition was obtained when the window size corresponded to the median bout length of that particular behaviour.

**Conclusions:**

In summary, the most effective strategy to significantly improve the accuracy of behaviour recognition is to use behaviour-specific parameters in the binary classification models.

**Supplementary Information:**

The online version contains supplementary material available at 10.1186/s12917-026-05294-1.

## Introduction

Computational techniques like machine learning (ML) and animal-borne motion sensors with accelerometers have significantly advanced behavioural science by enabling the collection and analysis of large-scale behavioural datasets —commonly referred to as ‘big behavioural data’ [1]. Among the various analytical approaches, machine learning has emerged as one of the most rapidly evolving and effective tools for processing the high volumes of sensor-derived data generated by accelerometers and other motion-tracking technologies [2].

The machine learning (ML) process consists of three main stages: the data preprocessing phase, the learning phase (which includes model training and evaluation), and the prediction phase (model deployment). While each stage influences overall model performance, prediction accuracy remains a primary metric for assessing a model’s effectiveness, particularly in behaviour recognition tasks, where precise classification is essential.

Data preprocessing plays a pivotal role in machine learning, as the quality of the input data directly affects the validity of conclusions. Effective preprocessing helps ensure that models analyse meaningful patterns rather than artefacts introduced by poor data handling. In behaviour recognition, the input is a sequence of time series data collected from sensors, which serves as the basis for the subsequent ML learning phase.

The basic scenario for using sensor data for behaviour recognition involves attaching a motion sensor to a dog to monitor its daily activities — such as walking, running, sleeping, eating, etc. The objective is to use machine learning techniques to automatically identify and classify these behaviours. Generally, there are two main approaches to processing sensor data for behaviour recognition:

1. Raw Data Processing with Machine Learning Models (Deep Learning Approach involves feeding the model raw sensor data without any manual feature engineering, allowing the machine learning algorithm—typically a deep learning model—to learn relevant patterns directly from the data. For example, in the context of dog behaviour tracking, a deep learning model could automatically distinguish between sleeping and playing based on the raw input, without requiring predefined rules or manually crafted features for each behaviour. In this approach, raw sensor data—such as accelerometer readings—is fed directly into machine learning algorithms without any initial preprocessing. Instead of manually preparing the data, the machine learning model- especially deep learning models [3, 4], such as Long Short Term Memory (LSTM) [5, 6] or Gated Recurrent Units (GRU) [6]) or Transformer models [7, 8] — learns to identify patterns on its own. These models are particularly effective at recognising temporal patterns—changes in data over time—similar to how they are used in natural language processing (NLP) to interpret sequences of words. However, deep learning models typically require large, well-labeled datasets to perform reliably. For instance, LSTM networks can learn to distinguish between behaviours such as running and sleeping by analysing time-based data from animal sensors.
2. The other approach is window-based segmentation, which is more commonly used in behaviour recognition. In this method, the continuous stream of raw sensor data is divided into smaller time windows or segments. For example, if an accelerometer records an animal’s movement over a 10-s interval, the data can be split into shorter windows—such as 1-s segments—each of which is independently analysed to classify specific behaviours (e.g., walking, eating, or resting). This approach is typically used with conventional machine learning models, such as Gradient Boosted Trees(GBT), including LightGBM [9] and XGBoost [10]. These models perform well when dealing with smaller datasets and less complex behavioural patterns. A sliding window technique, in particular, can help detect behavioural transitions—such as switching from foraging to resting—by capturing subtle changes in movement over short intervals.

Windowing methods in time-series data analysis can generally be classified into three main categories:

(i) In the Sliding Window Method (Fixed Time Intervals), the data is segmented into equal-length intervals, like taking a snapshot every few seconds, regardless of the animal’s actual behaviour at the time. For example, a dog’s movement may be recorded and analysed in 5-s windows, whether it is running, sitting, or jumping.
(ii) In the Event-Defined Window Method (Triggered by Specific Events), data segmentation is not based on fixed intervals but is instead initiated by the occurrence of an event, such as barking. Once the event is detected, the system extracts sensor data from a window that spans a specified period before and after the event (e.g., 5 seconds on either side) to provide contextual information and better understand the conditions leading to the behaviour.
(iii) In the Activity-Defined Window Method (Based on Movement Changes), data partitioning is based on detected changes in activity. A new segment is created each time the animal transitions from one behaviour to another. For example, when a dog shifts from walking to lying down, the system marks this change and defines a new segment accordingly. While event- and activity-defined windowing methods can yield behaviourally meaningful segments, a key challenge lies in accurately identifying the transition points or boundaries between behaviours.

Chakravarty *et al.* [11] addressed this issue by identifying characteristic micro-events—impulsive movements that generate brief (¡1 s) “shock signals” in accelerometer sequences collected from 10 free-living meerkats (Suricata suricatta) to detect four fine-scale foraging behaviours. These micro-events are visually recognisable patterns in the signal flow, like spikes in a graph, and could indicate a change in the behaviour. Their ‘seek-and-learn’ approach begins with detecting these micro-events by identifying peaks in the acceleration data envelopes (‘seek’), followed by the application of robust machine learning techniques (‘learn’) that extract meaningful features and distinguish between micro-events. The more frequently used fixed-length sliding window methods also have inherent pitfalls that can increase the redundancy and decrease the accuracy of the recognition. During the sliding process, consecutive windows may span two or more behaviours, introducing ambiguity. A more significant issue occurs when windows truncate the beginning or end of a behaviour, or include segments from adjacent behaviours [12]. This segmentation can result in mixed behavioural signals within a single window, which may confuse the model. Another challenge arises from the use of overlapping windows. The degree of overlap can influence ML performance [13]. When the overlap exceeds 50%, the same behaviour may be included in multiple windows, leading to overrepresentation of certain actions. For example, behaviours such as ”Walking” and ”Running” may be repeated too frequently, potentially biasing the model toward overpredicting these classes.

Even though the definitive role of the window length is broadly discussed and documented in the ML literature, it has contradictory results in behavioural research. Some studies assert that window size has no effect [12, 14, 15], while others have indicated that window length is important, exhibiting a slight [16, 17], or even strong [18] influence on the accuracy of the classification process. Barwick *et al.* examined the effects of sensor location and window length on the recognition examined the effects of sensor location and window length on the recognition show any significant effects on behaviour recognition across the four behaviours show any significant effects on behaviour recognition across the four behaviours approach to optimise the window length that performs best [19]. They often rely on values used in previously published studies without providing greater insight. studies without providing greater insight. The problem with this approach lies not only in its ad hoc assumptions, but in its reliance on using only one window size for feature ad hoc assumptions, but in its reliance on using only one window size for feature.

Considering these contradictory outcomes, our assumption is that cases where window length had no measurable effect on performance, could be attributed to a statistical problem. This occurs because the window size effect is masked when the performance outcomes of all behaviours are aggregated. At each specific window size, a mix of effective and less effective performers can be found, depending on how much the window size deviates from the optimal bout length of that behaviour.

As the data preprocessing phase is finished the second phase in ML process is the learning phase in which the model is trained to make the classification of the various behaviours. The learning part of the ML could run with or without human intervention and based on the human role three main directions are possible: unsupervised, supervised and a more recent ’reinforcement learning’. The main goal in unsupervised learning (UL) is to find similarities and differences between data points. Unlike supervised learning (SL) and reinforcement learning (RL), unsupervised learning does not require an agent to provide input on the correct set of actions (as in SL) or the rewards and punishments policy (as in RL).

So unsupervised machine learning models do not rely on input from a human observer. The main task of the algorithm is to find some ‘hidden’ structure in the data set by relying on various forms of clustering [20]. This results in statistically distinctive units that describe the whole data set. For example, this method is used by neurologists who aim to reconstruct the temporal pattern of the behaviour down to the smallest detail, to understand how neural networks control behaviour [21]. Their approach used by neurologists is highly reductionist [22], focusing solely on the temporal pattern of the behaviour. While this focus can be sufficient for predicting underlying neural mechanisms of behaviour, it entirely overlooks the function of the actual behaviour which is at the heart of an ethological analysis.

In supervised learning, such as behaviour recognition by machine learning, the raw data are labelled by human observers after achieving high inter-observer reliability., The supervised ML algorithm is then trained to recognise specific behaviours. If this training process is successful, and the sampled data is sufficiently large and representative, then the ML method can achieve recognition performance comparable to or better than that of a human observer. Nevertheless, the accuracy of the ML method depends on the input provided by the human observer [23, 24]. In this article, the supervised ML method is used as this is the most frequently employed method in behavioural science, providing ethologists and psychologist with the greatest ability to influence the behaviour recognition process.

Reinforcement learning [25] is a relatively new method in ML and can cause confusion due to its similarity to the biological term widely used for animal and human learning in ethology and psychology, therefore, it is more precise to refer to it as computational RL. Computational RL methods in machine learning are a good example for using a mathematical method to simulate and model a biological phenomenon like reinforcement learning. The better the model’s predictions and the closer its algorithm is to its real-world counterpart, the closer we come to understanding the biological phenomenon [26].

A more ambitious goal is to combine the three divergent approaches (UL, SL and computational RL) into ML models for behaviour recognition. These models would increasingly build on the internal regulation of behaviour and incorporate the biologically determined and defined functions. This is especially true for the computational RL, as RL tries to model the reinforcement learning of animals and humans. An ML model performs better if its operation aligns as close with the biological regulation of the behaviour as possible. By working as a scientific model of behaviour organisation, it can make more accurate predictions and recognise behaviours more efficiently. This phenomenon is similar to the concept of representational equivalence between deep neural networks (DNN) and the primate brain [27], or the examination of early visual representations from the retina to the cortex using anatomically constrained deep convolutional neural network (CNN) [28]. As Cichy asked in his article [29] about Deep Neural Networks as Scientific Models: “Can the fit between DNNs and biological systems be improved by infusing DNNs directly with neural or behavioural data? Will such methods yield DNN models that more closely resemble the human brain and better predict successes and errors in human behaviour?”

One more consideration to be made in case of a supervised learning task in the learning phase is whether the classification algorithm should be Multi-Class or Multi-Label. For behaviour recognition, the model has to choose only one class from all possible behaviours for a given time segment(window), making Multi-Class to be the evident choice. It is trivial that in case of Multi-Class ML methods the same window size must be used for all behaviour, as only one model is employed for recognising all behaviours. On the other hand, every Multi-Class methods can also approach a multi-class classification problem by dividing into multiple binary classification tasks, where a separate binary classification model is trained for each behaviour. This approach requires the model to determine whether a given time segment (window) belongs to a specific behaviour or not, this is why it is referred to as One-vs-All (OvA) [30]. In this case as every behaviour recognition has a different model and every model can use different window sizes. As our hypothesis was that every behaviour recognition model has a different optimal window size OvA ML model was used.

Ethologists break up the continuous flow of behaviour sequence (animal motion) into simpler behaviours [31], which are discrete building blocks of the species-specific behaviour repertoire sharing the same movement pattern and function. These building blocks, known as the ethogram, are used to quantify the behaviour of the subject during observation. For such segmentation of behaviour movements, it must be determined when a specific behaviour starts and ends. Behaviours based on a specific movement pattern are separated in the temporal analysis of animal behaviour sequences, leading to the identification of a well-known phenomenon called ’bout’ [32]. The bout is defined as a homogeneous, temporally clustered behaviour without interruption from its start to its finish [33]. Behaviours can be characterised by typical bout lengths, which in some specific cases may reflect behavioural or physiological constraints. For example, the breathing patterns of whales seem to be related to the lung capacity of the animal [34]. The analysis of grooming behaviour in mice provides extensive and detailed data on the genetic determination of bout length. The examination of different mouse strains revealed that they displayed varying durations of grooming, suggesting the possibility of a strong genetic influence [35]. Stereotypical behaviours also exhibit specific bout lengths. For example, in children with Autism Spectrum Disorder (ASD), certain stereotyped behaviors are not only more common but also last longer than in their typically developing peers [36]. Typically, the most documented behaviours with specific bout lengths play a role in foraging, grooming, loco-motor, sleeping, and anti-predator behaviour [37–39].

Interestingly, separating behaviour into bouts or cutting the sequence into smaller time interval chunks are conceptually analogous to ML window segmentation. Our hypothesis is that for each behaviour, there should be an optimal window length for its recognition, and this window size may differ for each behaviour. Therefore, using a binary classification model with different window sizes for each behaviour would be more appropriate than a multi-class model that uses the same window size for every behaviour. In this study, we test the hypothesis that using a binary class model with behaviour-specific window sizes is more effective than using a multi-class ML model with a uniform window size for all behaviours. Additionally, we aim to find evidence suggesting that the optimal window size could align with the biologically determined bout length of the given behaviour.

## Materials and methods

### Subjects

In this research dogs were use as the focal experimental species due to their wide range of behavioural repertoires. Our subjects were 56 healthy adult family dogs, consisting of 31 purebreds from 17 different breeds and 25 mixed breeds. The group included 26 males and 30 females, with a mean age of 5.9 years (sd = 3.3), and a mean weight of 20.7 kg (sd = 8.08). Owners accompanied their dogs to the university premises from their homes in Budapest, Hungary, or within close proximity of Budapest. Prior to the experiment, all dogs underwent a veterinary examination, which included assessment of body condition, skin, coat, eyes, ears, teeth, and weight. All dogs were confirmed to be in good physical health and free from any orthopaedic or neurological disorders [40].

### Data collection devices

**Fig. 1:**
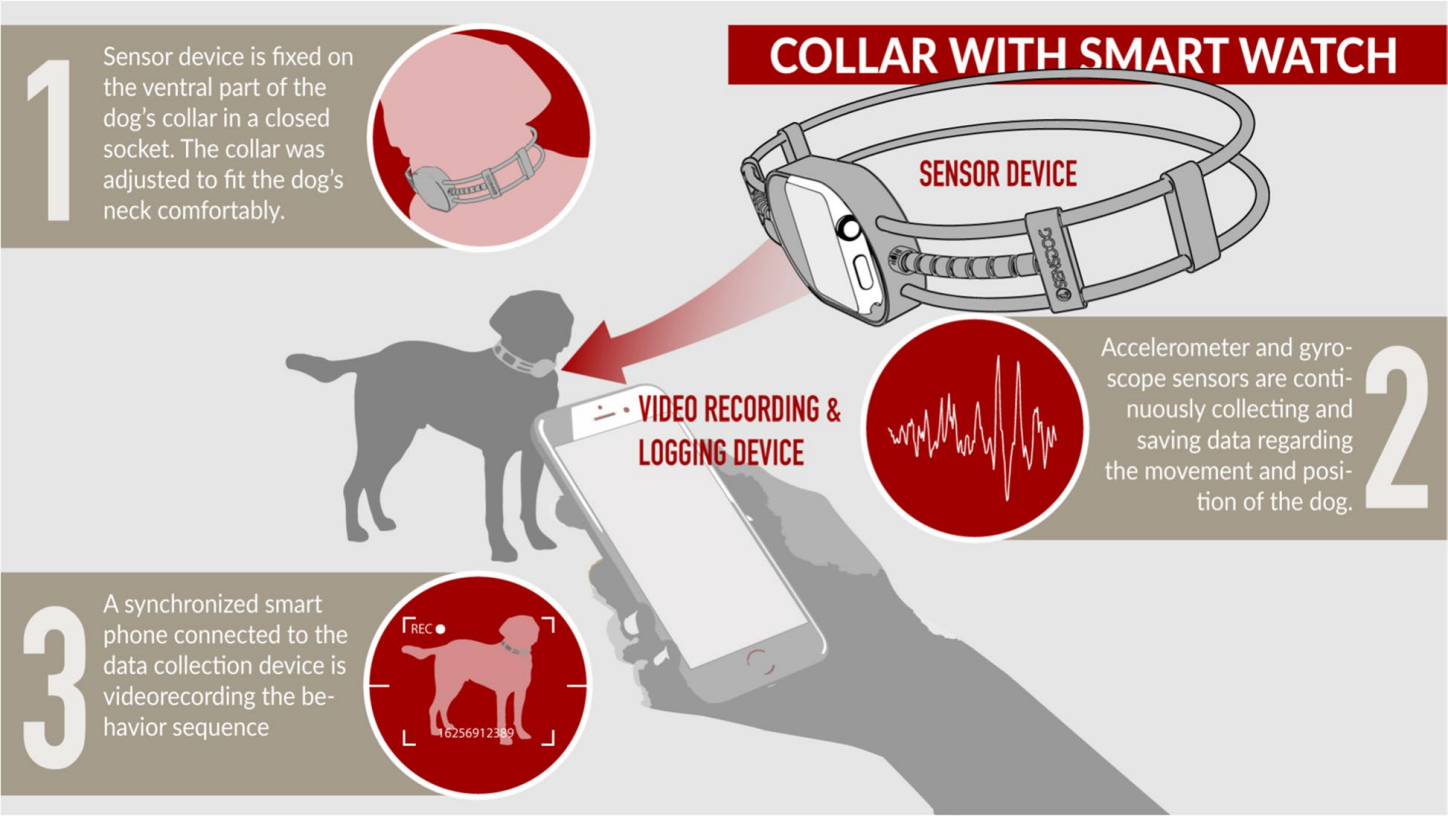
Data collection method with SensDog

The data collection equipment, which was used in previous experiments as well-consisted [40] of two devices: one sensor device and one video recording and logging device, both running the SensDog software (see Fig. 1). As a sensor device an Apple Watch was used, which is a commercially available Apple product. The SensDog software is a member of the SensX software family, which is under development and not yet commercially available. The sensor device contained a 9-axis motion sensor (3-axis gyroscope + 3-axis accelerometer + 3-axis magnetometer) sampling data at 50 Hz (50 sample/sec). In the measurement each sample data point contained 3 dimensional parameters of the device (x, y, z axis) for rotation and acceleration, and 4 dimensions for attitude, referring to the amount of rotation around each of the axes of the device(using quaternion, with x, y, z, w axis). During the experimental session the sensor device processed and stored the gyroscope and accelerometer data in real time. At the end of the session, the processed sensor data were sent to the Bluetooth-connected logging device, which simultaneously recorded the session on video and then transferred the data and video to our network servers. The synchronisation of the raw data and the video was made automatically by the SensDog software.

### Data collection protocol

The measurements were conducted in indoor (*N* = 13) or outdoor (*N* = 43) settings. Indoor observations took place at the department with ample space (26mx3m, 78 m2, with vinyl flooring and with window on one side) for the dogs to move freely. Outdoor observations were carried out in an area with plain, grassy, and even surface around the university campus without fence. Each participating dog was fitted with our sensor device before the start of the session. The sensor device was securely housed in a closed socket that was attached on the ventral part of a specially designed collar. The collar was adjusted to fit the neck of the dog comfortably as shown in Fig. 1. A side view of the dogs wearing the sensor device was video recorded at all times throughout the experiment. Data logging on the inertial data logger was initiated simultaneously with start of the video recording. The dogs were allowed to move freely during the experiment. Owners were instructed to minimize interruptions and avoid direct contact with their dogs as much as possible.

Intervention was allowed in cases when a specific behaviour—like sitting—was not represented in the behaviour sequence. There were seven obligatory behaviours. The definitions of the behaviours are summarised in Table 1. The data collection process focused on these behaviours and it was recorded for 12–25 min. For these induced behaviours, the owner has no influence on the pace or termination of the behaviour. Behaviours not listed were also recorded and coded but not used in this research.

**Table 1:** Non-overlapping behaviours of dogs used in the classification process

Short name	Description
Stand	The animal stays in one place with four straight legs touching the ground. Movements of the tail and the head are allowed.
Sit	The animal stays in one place with front legs in extended position, both hind legs bent, and the metatarsal regions touch the ground. Movements of the tail and the head are allowed.
Lie	The animal stays in one place in sternal recumbency with all four legs bent and both elbows touching the ground. Movements of the tail and the head are allowed.
Eat	The jaw of the dog is close to a specific food source and is ingesting (chewing and swallowing) food.
Walk	The animal is using the slowest, symmetric four-beat gait, by which it supports its weight with its feet in the following sequence: front left limb, hind right limb, front right limb, hind right limb
Trot	The animal is performing a symmetric gait by which the diagonal pairs of limbs move almost simultaneously.
Run	The animal is performing an asymmetrical, three- (i.e. canter) or four-beat (i.e. gallop) gait. During canter, the gait pattern is hind foot, the opposite hind foot and its front diagonal, followed by the other front foot and suspension, when present. In gallop the animal supports its weight with its feet in the following sequence: right front leg, left front leg, right hind leg, left hind leg

### Data preparation and labelling

As supervised machine learning algorithms were used it was a prerequisite to prepare the behaviour labels for teaching the ML model accordingly. Data segments from the time series sensory measurements were synchronised with the video recordings and labelled by a human observer. The data preprocessing consists of three main parts: the video coding, the bout length analysis and the feature extraction.

#### Video coding

Solomon Coder (©Andr´as P´eter) was used for video coding. The coding protocol included definitions of 8 behaviour (see Table 1). Video recordings were coded by five coders and the labels were automatically synchronised to the IMU(Inertial Measurement Unit) data by the SensDog software. The coders were trained based on a standardised protocol used by the Ethology Department, ELTE Budapest. The ethogram of the eight dog non-overlapping behaviours were used for classification. A total of 2,767,500 (1/50 s) sample points of video-labelled behaviour were collected from 56 dogs.

#### Bout length analysis

After coding, a label from the manual labelling was assigned to each sample point in the time series of the sensor data. Based on these labels, the exact bout lengths of the various behaviours were calculated.

#### Feature extraction

The sensor device recorded data across 13 channels: acceleration (x,y,z), gravity (x,y,z), rotation (x,y,z), and attitude (x,y,z,w as a quaternion). Additionally, three derived channels were added:and it was added 3 more channels: acceleration x/z ratio, acceleration absolute value, rotation rate absolute value, totaling 16 channels. The gradients of these 16 channels were also calculated, resulting in 32 channels (16 original + 16 gradients). For each window, 5 statistics resulting in 32 channels (16 original + 16 gradients). For each window, 5 statistics resulting in 160 features (32 channels * 5 statistics).

### Data analysis

An implementation of Gradient-Boosted Trees (GBT), specifically LightGBM [9] (Light GBT) was selected for categorisation because our previous research [41] on other datasets demonstrated that LGBM delivers the best performance. It significantly outperformed other boosted tree methods, such as XGBoost (XGBT) [10] in terms of speed and computational efficiency, reducing computational time per iteration by 85%. In comparison, recurrent neural networks, such as Long Short Term Memory (LSTM) [5] and Gated Recurrent Unit (GRU) [6], can learn patterns with high accuracy when dealing with a large number of sequences. However, in our case—consistent with findings from our previous research— recurrent neural networks did not match the performance levels achieved by the GBT methods. Some behaviours (e.g.,”walking”) occurred far more frequently than others (e.g.,”eat”). This imbalance can lead the machine learning model to prioritise the more common behaviours while underperforming on the rarer ones. To address this issue, we employed SMOTE(Synthetic Minority Over-sampling Technique), a method that generates synthetic examples of underrepresented behaviours to ensure the model learns to recognise all classes more effectively [42]. A method called cross-validation (CV) was used, in which the dataset was randomly shuffled and divided into smaller subsets. In each iteration, the model was trained on a portion of the data and tested on the remaining subset. This approach helped ensure that the model was learning generalisable patterns rather than simply memorising specific examples. So, to train and evaluate the ML model, the data set was separated into three independent data sets for training, validation and for testing. On the training and validation set k-fold cross-validation (CV) process was used to randomly partition the dataset into k equal subsets. The model was trained on k-1 subsets, and the remaining subset was used for validation. In case of a threefold CV method in each epoch the 25 + 25% of the dataset was used for training and 25% of the dataset was used for the validation purposes. The validation set was used to tune the hyper-parameters and check for overfitting errors. Then the separated remaining 25% test set was used to evaluate the model’s performance. We used cross-validation in a way that ensured the training and test sets did not include behaviour sequences from the same dog. Therefore, this method is referred to as subject-independent CV.

One of the most important statistical assumption for ML processes is that samples are independent and identically distributed (i.i.d.), that is, all the data points are sampled independently from the same distribution. However, samples drawn from the same subject are most likely not independent. This means that the similarity of samples drawn from the same participants is likely to be higher than the samples drawn from different participants (see also [43]). This kind of bias of k-fold CV may overestimate the performance of categorisation by relying on correlations within subjects [43]. This problem of k-fold CV is more serious when it is used with overlapping sliding windows, because these overlaps between adjacent windows are another source of unwanted dependency between data points.

To address these issues the training, validation and testing sets were split by dogs. It means that different data sets from different individuals were used for training, validating and testing purposes. According to this method, in each iteration, the model is trained on all the participants except those, which are used for testing. In our case, we separated the participants into 3-folds using 2-folds for training and one-fold for testing.

**Fig. 2:**
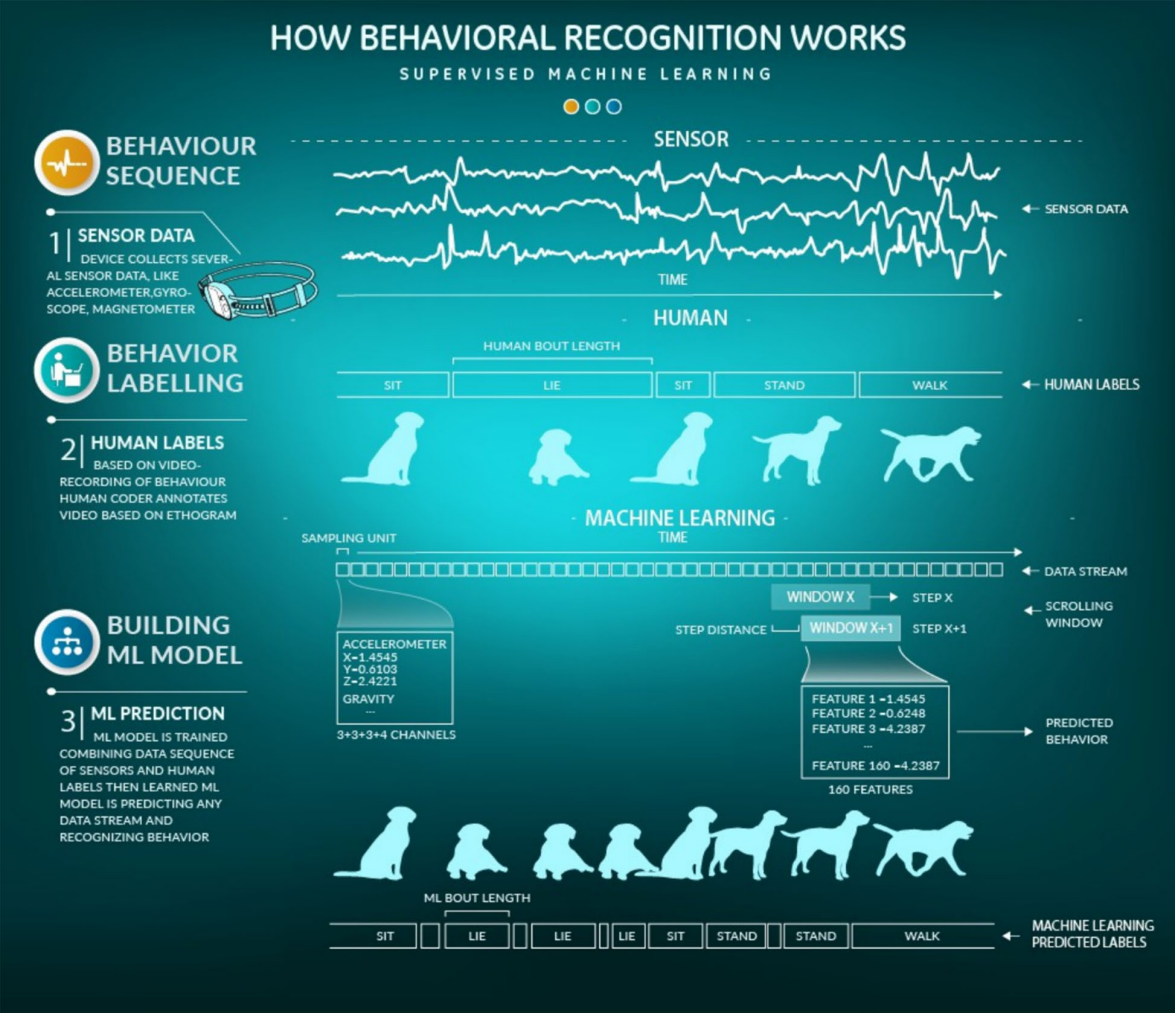
The video annotation and sensor data preparation for the supervised machine learning method used. Each sampling unit contains data from 13 sensor channels. Depending on the window size, each window comprises a specified number of these sampling units, from which 160 features are calculated. After each calculation, the window shifts along the timeline by 5 sample points

We used the dynamic overlapping sliding window technique for data segmentation with a specified window length, scrolling the window 10 sampling units forward, but no less than 5 sample units in the case of very small windows like 15 and 32. Sliding window sizes of 15, 32, 60, 81, 100, 149, 200, 300 sample points (0.3, 06, 1.2, 1.6, 2.0, 2.9, 4, 6 s) were considered for feature computation; this provided sufficient temporal resolution of the behaviour and was short enough to capture bouts of activities with the shortest duration. Successive windows had an overlap of 5 sample points. Windows containing transitions between different activities were labelled as the behaviour at the end of the bout. Thus, each window is assigned to only one behaviour, determined by the last sample point of the window, even if multiple video-labeled behaviours(transitions from one behaviour to another) are present within the window. The process of the method is summarised in the Fig. 2.

As any multi-class problem could be built up from binary classifications, separate binary models were used instead of one multi-label model. One model was used for each behaviour separately comparing the positive class to the remaining classes combined. For each behaviour, 10 iterations per window size per fold were conducted, resulting in 240 total runs. To evaluate how well our AI model recognised dog behaviours, we used a score called AUC (Area Under the Curve). This number ranges from 0.5 to 1.0—the higher, the better. The weighted AUC value (0.5–1.0) of the run of the behaviour was calculated as the indicator of recognition success. The ROC AUC score is the area under the ROC curve (Receiver Operating Characteristic curve). The ROC curve shows the performance of a binary classifier with different decision thresholds.

The effect of window size on performance measured in AUC was estimated by 2nd order polynomial regression model. The *p* value of the statistical models was used to decide how well the 2nd order polynomial regression fits and based on this regression function the local maximum was determined. As there are several factors that affect the AUC values of the ML model and the window size is only one of these factors, the errors of the regression models which caused the low *R*^2^ values was not examined further.

## Results

### Bout length of the behaviours

Each behaviour was characterized by its median bout length, calculated from the coding data as the uninterrupted median length from its start to its finish for every occurrence of that behaviour. The median of the bout lengths ranged from 62–365 sample units, equivalent to 1.22–7.3 s. Each sample unit corresponded to one sample set of the inertial sensor data collected every 20 ms (50 Hz). For each behaviour the mean/median/std of the bout length was computed (Table 2). The behaviours with shorter bout length, like walk, trot, and run were frequently interrupted by other activities. Passive behaviours, like Stand, Sit and Lie, as well as Eat typically manifested in longer bout length. The number of occurrences of behaviours collected varied from 72 to 3957 (see length. The number of occurrences of behaviours collected varied from 72 to 3957 (see Table 2). The frequency distribution of bout lengths of the behaviours is illustrated in the figures from S15 to S21.

**Table 2:** Summary statistics of bouts of the behaviours (in sample units, rate 1/50 s)

Behaviour	Bout length in sample unit			Number of occurrences of bouts	2nd order regression high (sample units)	Difference from bout median (sample unit)
	median	mean	std			
Stand	77	197	596	2952	65	−12
Sit	174	372	801	540	138	−36
Lie	293	664	2657	394	249	−44
Eat	366	785	810	72	300	−66
Walk	62	170	547	3932	83	21
Trot	85	130	151	1406	79	−6
Run	66	100	117	1115	88	22

### Classification performance of specific activities by window sizes

**Fig. 3:**
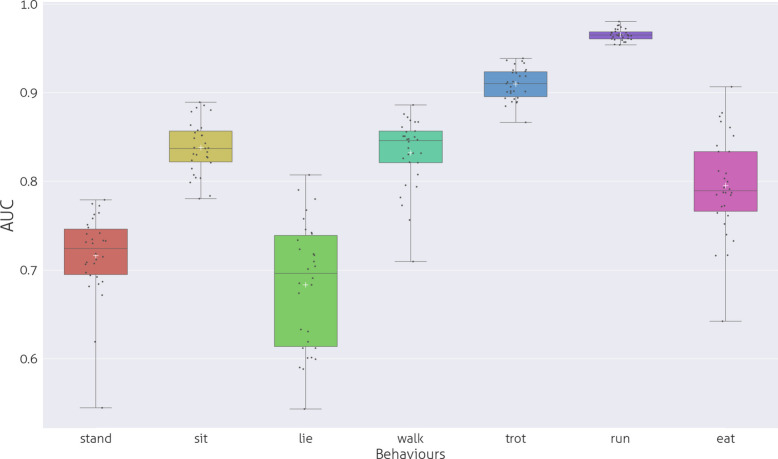
Performance of the ML recognition by behaviours in AUC

The overall performance of the LGBM model regardless of window size, adding up the results of all measurements, ranged from AUC 0.6962 (“Lie”) as the lowest to AUC 0.9828 (“Run”) as the highest (Fig. 3).

The performance of the LGBM model with default parameters regarding the recognition of behaviours as a function of window size is showing on Table S1-S7 and on Fig. S8-S14.

**Fig. 4:**
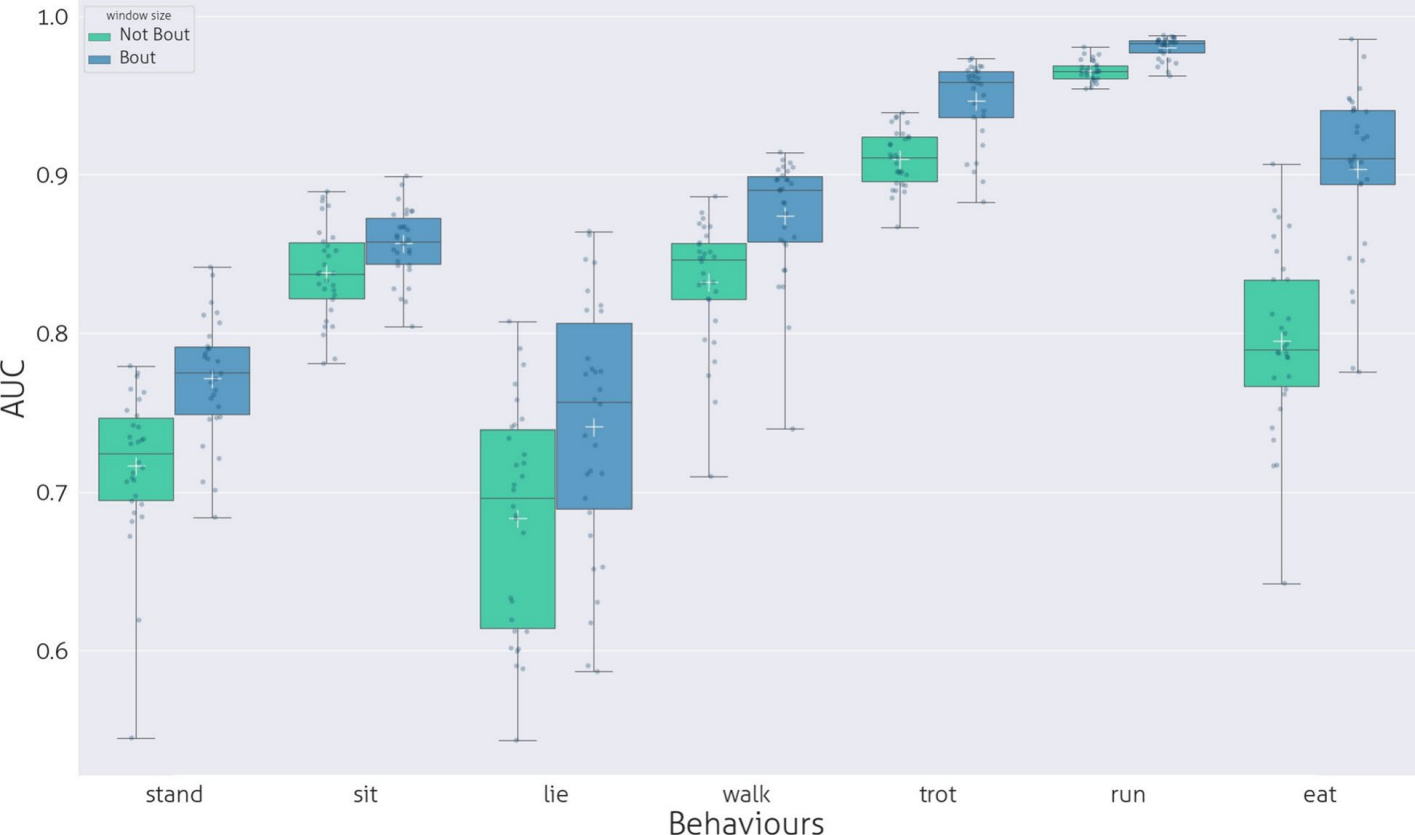
Comparison of Performance of the ML recognition of behaviours in AUC with”no bout length window” and”bout length window”

In the case of behaviours that have longer or similar bout length than the maximum In the case of behaviours that have longer or similar bout length than the maximum In the case of behaviours that have longer or similar bout length than the maximum toward the median bout length, reaching a maximum around the bout length. If the bout length median is smaller than the maximum window size, the maximum of the polynomial is around the bout length median (see Fig. S8-S14. The highest value of R2 was 0.323 for “Run” with p ¡ 0.001, while the lowest was R2 = 0.071, p ¡ 0.001 for “Lie”. In the graphs in Fig. S8-S14 the fitting of the polynomial regression analysis is shown. The local maxima of the data (the most effective window length) are where the AUC values are the best, close to the median bout length. The difference between the classical ML windowing methods is displayed in Fig. 4, which employs the same window length for all binary classifications, and our proposed method, which utilises a window size closest to the bout length of the specific behaviour. The decrease in AUC values is influenced by the extent to which the window size deviates from the median bout length of the behaviour. If the bout median falls within the range of 293–366 samples, the farthest value is 15. For the range of 62–85 samples (Walk, Run, Stand, Trot), the farthest value is 300. The sit bout value is 1744 samples, and 300 was selected as the farthest window size.

## Discussion

In this study, we present a novel approach to behaviour recognition using machine learning by aligning the sliding window size with the median duration of each specific behaviour. Traditionally, machine learning models employ a fixed window length for segmenting data [44], often determined arbitrarily or based on prior heuristics. However, our hypothesis was that that aligning the window size with the natural length of behaviours would improve recognition accuracy. To the best of our knowledge, this study is among the first to systematically investigate the use of behaviour-specific bout lengths as a biologically informed parameter for selecting window size in machine learning-based behaviour recognition.

The results of our analysis provide strong support for this hypothesis. By incorporating a machine learning parameter that reflects the typical duration of a behaviour, we enhanced the model’s ability to detect and classify behaviours with greater accuracy. Specifically, our experiments using the LightGBM (LGBM) binary classification model revealed that window size significantly impacts recognition performance. The optimal recognition accuracy, measured using the AUC (Area Under the Curve) score, varied across different behaviours. However, in all cases, the AUC value peaked when the window size closely matched the median bout length of the respective behaviour.

This finding has important implications for behaviour recognition models. It suggests that a one-size-fits-all approach to window selection is suboptimal. Since different behaviours have distinct durations, each behaviour requires its own optimal window size to achieve best performance. Using a single fixed window length across all behaviours—as is common in many machine learning models – can result in segments that either truncate individual behaviours or overlap between different behaviours, ultimately reducing model accuracy.

To address this, we found that binary classification models, where each behaviour is detected separately with its own optimised window size, provide a more precise solution. In contrast, multi-class classification models, which use a single window size for all behaviours, are inherently limited because they cannot adjust to the varying durations of different behaviours. This limitation can lead to misclassification, as a window length that is suitable for one behaviour may be too short or too long for others, thereby reducing overall recognition performance. By dynamically selecting window sizes based on behaviour duration, our method significantly improves the AI’s ability to recognise behaviours with high accuracy. This approach represents a step forward in optimising machine learning techniques for behaviour analysis, ensuring that models are better aligned with the natural characteristics of the behaviours they aim to recognise.

Although our method could provide a practical guideline and starting point to build better behaviour recognition ML models, we have to take into consideration that the biological processes underlying the bout length of a particular behaviour could be very different. There are behaviours (e.g. modal action patterns) that have a more strictly defined temporal structure, for which the bout length is closely controlled by physiological processes. There are certain types of behaviours in which an internal neural system determines the rhythm, e.g., the wing flap rhythm of flies [45], or the visuomotor activity in zebrafish larvae [46]. The Central Pattern Generator is a good example of determining the bout length of a set of behaviours that form a repeated pattern, like the grooming behaviour in Drosophila [22].

The behaviour of freely moving dogs is determined by many internal and external factors, and the bout lengths of many behaviours are very variable. Although the flexibility of a particular behaviour can also present an evolutionary advantage [47], we still find that bout length is relatively stable. In species with variable morphology, like dogs, it may be especially important to determine the bout length, for example at the level of the breed because smaller animals change their behaviour more often, at much shorter intervals than larger ones.

There is one practical consideration: the bout of a given behaviour has to be homogeneous and the coding has to be very precise regarding the starting and ending point of the bout and even the smallest interruption has to be coded. The frequency distribution of our data shows this precision as there are bout lengths with sub-second intervals. The other important factor that when ML model teaching is the aim it is important to care with the homogeneity of the definitions of the behaviour regarding the special requirements of the sensor and using these definitions strictly. For example, if the code is”Walk” as a given behaviour, we have to separate the walking in one direction from the turning as during the turning the gyroscope is activated as the turning movement of the body is different in the data stream from the walk data pattern so the ML model could learn the difference and the accuracy of the behaviour recognition is much better.

In our implementation, the one-vs-all models are combined at inference time by comparing their predicted probabilities, so that a single behaviour label is produced for each time segment. We note, however, that maintaining multiple window sizes and classifiers can become operationally demanding for large behaviour vocabularies and is therefore best viewed as a proof-of-concept for an ethologically motivated, window-based preprocessing strategy. The same bout-length–based choice of window size could also be used to define temporal segments for downstream unsupervised or self-supervised representation learning, without being tied to a specific supervised classifier.

So, our proposed method for using bout length in developing ML model is as follow: collecting video recording of the various behaviours of the given species and meantime collect data of the movement with an inertial sensor, then assign behaviour labels to this data stream. Then define the bout length of the behaviours one by one and teach our proposed supervised ML method for every behaviour as a binary classification using the median of the bout lengths of the given behaviour as the window size. With this method a much more accurate ML model can be developed, and this ML model can be used in experiments for any task, avoiding the cumbersome manual coding in the future.

As a conclusion it can be stated that it is always worthwhile to build an ML model for behaviour recognition that corresponds to the biologically determined properties of the specific behaviour, rather than trying to optimise ML parameters by working with fictitious numbers. The ML method is more effective if it is developed as specifically as possible for a particular behaviour. Combining these specific models into a higher-level hierarchical supermodel that attempts to approximate the hierarchical structure of behaviour could be a good practice. Behaviour-specific binary ML models can incorporate models developed by neurobiologists, which are based on highly detailed temporal patterns, to create behaviour recognition models that take into account the functionality of behaviour advocated by ethologists.

## Conclusion

Behaviour sequences were collected using a special SensDog software, with a smartwatch attached to the collar of the dogs, allowing them to move freely. We used a Light Gradient Boosted Machine (LGBM) learning algorithm, with a threefold cross-validation in a binary classification task. We applied a sliding window technique for signal processing, and we analysed the effect of window size on recognition performance. Although all activities were successfully recognised with AUC values above 0.7, using a biologically relevant parameter, bout length of behaviours, increased the performance. We conclude that bout length provides a good default value for choosing the optimal window length for ML models used to recognise behaviour. So, our proposed method for using bout length in developing ML model is simply: after video-recording and collection inertial sensor data and annotate behaviour labels, define the bout length of the behaviours one by one and teach our proposed supervised ML method for every behaviour as a binary classification using the median of the bout length of the given behaviour as the window size. With the bout lengths in mind a much more accurate ML model could be developed using a binary ML models to avoid the cumbersome manual coding in the future.

Overall, our results indicate that bout length provides a biologically interpretable and practically useful guideline for choosing window sizes in the data-preparation phase. We do not propose bout-length–based segmentation as a long-term solution for all behaviour recognition tasks or as a substitute for modern sequence models; rather, we see it as a simple, ethological prior that can inform window selection before both supervised and unsupervised or self-supervised learning. In the longer term, we expect scalable self-supervised and hierarchical approaches that internalise multi-scale temporal structure to play a central role, and our findings may inform how such models are initialised, regularised or interpreted.

## Supplementary Information

Supplementary Material 1.

## Data Availability

The source code used in the current study is available in the GitHub repository, (https://github.com/BootMaker/Bout_ML_behaviour_2024) The complete datasets used and analysed during the current study are available from the corresponding author on reasonable request.
