# Supplementary material for: Recognising dog movement with behaviour-specific machine learning models: bout length as a biologically relevant parameter for window size
Source: BMC Vet Res. 2026 Mar 19;22:248. doi: 10.1186/s12917-026-05294-1 (PMC13123084; doi:10.1186/s12917-026-05294-1)
Supplement: Supplementary file 1 — Supplementary Material 1. [file 12917_2026_5294_MOESM1_ESM.docx]

**Appendix** 556

**A1 Table. Lie measurement data** The AUC median and std values of the LGBM 557

models by window lengths for the ”Lie” behaviour 558

| **Label** | **Window size** | **Count** | **AUC median** | **Std** |
| --- | --- | --- | --- | --- |
|  | 15 | 30 | 0.696 | 0.071 |
|  | 32 | 30 | 0.706 | 0.068 |
|  | 60 | 30 | 0.729 | 0.069 |
| lie | 81 | 30 | 0.727 | 0.065 |
|  | 120 | 30 | 0.723 | 0.072 |
|  | 149 | 30 | 0.720 | 0.645 |
|  | 200 | 30 | 0.753 | 0.061 |
|  | **300** | **30** | **0.757** | **0.080** |

**A2 Table. Sit measurement data** The AUC median and std values of the LGBM 559

models by window lengths for the ”Sit” behaviour 560

| **Label** | **Window size** | **Count** | **AUC median** | **Std** |
| --- | --- | --- | --- | --- |
|  | 15 | 30 | 0.837 | 0.029 |
|  | 32 | 30 | 0.850 | 0.028 |
|  | 60 | 30 | 0.851 | 0.031 |
| sit | 81 | 30 | 0.850 | 0.029 |
|  | **120** | **30** | **0.858** | **0.022** |
|  | 149 | 30 | 0.846 | 0.028 |
|  | 200 | 30 | 0.853 | 0.027 |
|  | 300 | 30 | 0.837 | 0.034 |

**A3 Table. Stand measurement data** The AUC median and std values of the 561

LGBM models by window lengths for the ”Stand” behaviour 562

| **Label** | **Window size** | **Count** | **AUC median** | **Std** |
| --- | --- | --- | --- | --- |
|  | 15 | 30 | 0.757 | 0.041 |
|  | 32 | 30 | 0.773 | 0.033 |
|  | 60 | 30 | 0.774 | 0.039 |
| stand | **81** | **30** | **0.775** | **0.038** |
|  | 120 | 30 | 0.765 | 0.040 |
|  | 149 | 30 | 0.756 | 0.036 |
|  | 200 | 30 | 0.747 | 0.045 |
|  | 300 | 30 | 0.725 | 0.048 |

**A4 Table. Eat measurement data** The AUC median and std values of the LGBM 563

models by window lengths for the ”Eat” behaviour 564

| **Label** | **Window size** | **Count** | **AUC median** | **Std** |
| --- | --- | --- | --- | --- |
|  | 15 | 30 | 0.790 | 0.056 |
|  | 32 | 30 | 0.844 | 0.044 |
|  | 60 | 30 | 0.857 | 0.065 |
| eat | 81 | 30 | 0.859 | 0.072 |
|  | 120 | 30 | 0.872 | 0.060 |
|  | 149 | 30 | 0.879 | 0.077 |
|  | 200 | 30 | 0.877 | 0.052 |
|  | **300** | **30** | **0.910** | **0.053** |

**A5 Table. Walk measurement data** The AUC median and std values of the 565

LGBM models by window lengths for ”Walk” behaviour 566

| **Label** | **Window size** | **Count** | **AUC median** | **Std** |
| --- | --- | --- | --- | --- |
|  | 15 | 30 | 0.851 | 0.040 |
|  | 32 | 30 | 0.878 | 0.031 |
|  | 60 | 30 | 0.884 | 0.027 |
| walk | **81** | **30** | **0.890** | **0.038** |
|  | 120 | 30 | 0.870 | 0.034 |
|  | 149 | 30 | 0.861 | 0.042 |
|  | 200 | 30 | 0.851 | 0.028 |
|  | 300 | 30 | 0.846 | 0.004 |

**A6 Table. Trot measurement data** The AUC median and std values of the LGBM 567

models by window lengths for ”Trot” behaviour 568

| **Label** | **Window size** | **Count** | **AUC median** | **Std** |
| --- | --- | --- | --- | --- |
|  | 15 | 30 | 0.936 | 0.020 |
|  | 32 | 30 | 0.951 | 0.019 |
|  | **60** | **30** | **0.958** | **0.026** |
| trot | 81 | 30 | 0.957 | 0.019 |
|  | 120 | 30 | 0.951 | 0.023 |
|  | 149 | 30 | 0.940 | 0.017 |
|  | 200 | 30 | 0.927 | 0.019 |
|  | 300 | 30 | 0.910 | 0.018 |

**A7 Table. Run measurement data** The AUC median and std values of the LGBM 569

models by window lengths for ”Run” behaviour 570

| **Label** | **Window size** | **Count** | **AUC median** | **Std** |
| --- | --- | --- | --- | --- |
|  | 15 | 30 | 0.972 | 0.006 |
|  | 32 | 30 | 0.978 | 0.005 |
|  | **60** | **30** | **0.983** | **0.007** |
| run | 81 | 30 | 0.980 | 0.005 |
|  | 120 | 30 | 0.980 | 0.007 |
|  | 149 | 30 | 0.976 | 0.005 |
|  | 200 | 30 | 0.974 | 0.005 |
|  | 300 | 30 | 0.965 | 0.006 |

# A8 Fig. Performance of the machine learning classification model by 571

**window length in case of ”Lie”.** A 2-order polynomial curve fitted to the AUC 572

values of ”Lie” by window sizes. The blue vertical line shows the bout length median 573

(249), R2=0.071, p=0.000. The data points contain a horizontal jitter for better 574

visuality. 575


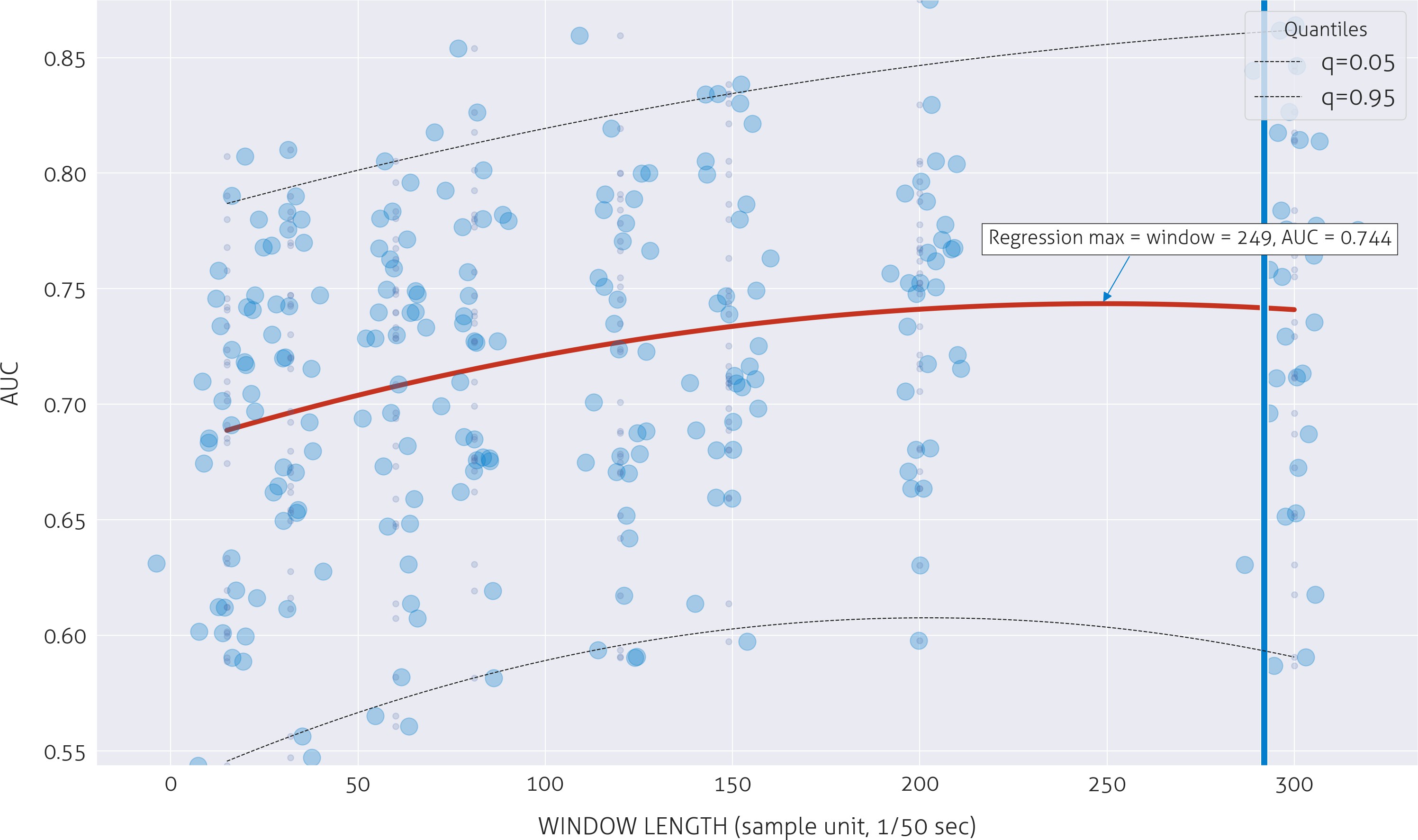


576

# A9 Fig. Performance of the machine learning classification model by 577

**window length in case of ”Sit”.** A 2-order polynomial curve fitted to the AUC 578

values of ”Sit” by window sizes. The blue vertical line shows the bout length median 579

(138), R2=0.062, p=0.001. The data points contain a horizontal jitter for better 580

visuality. 581


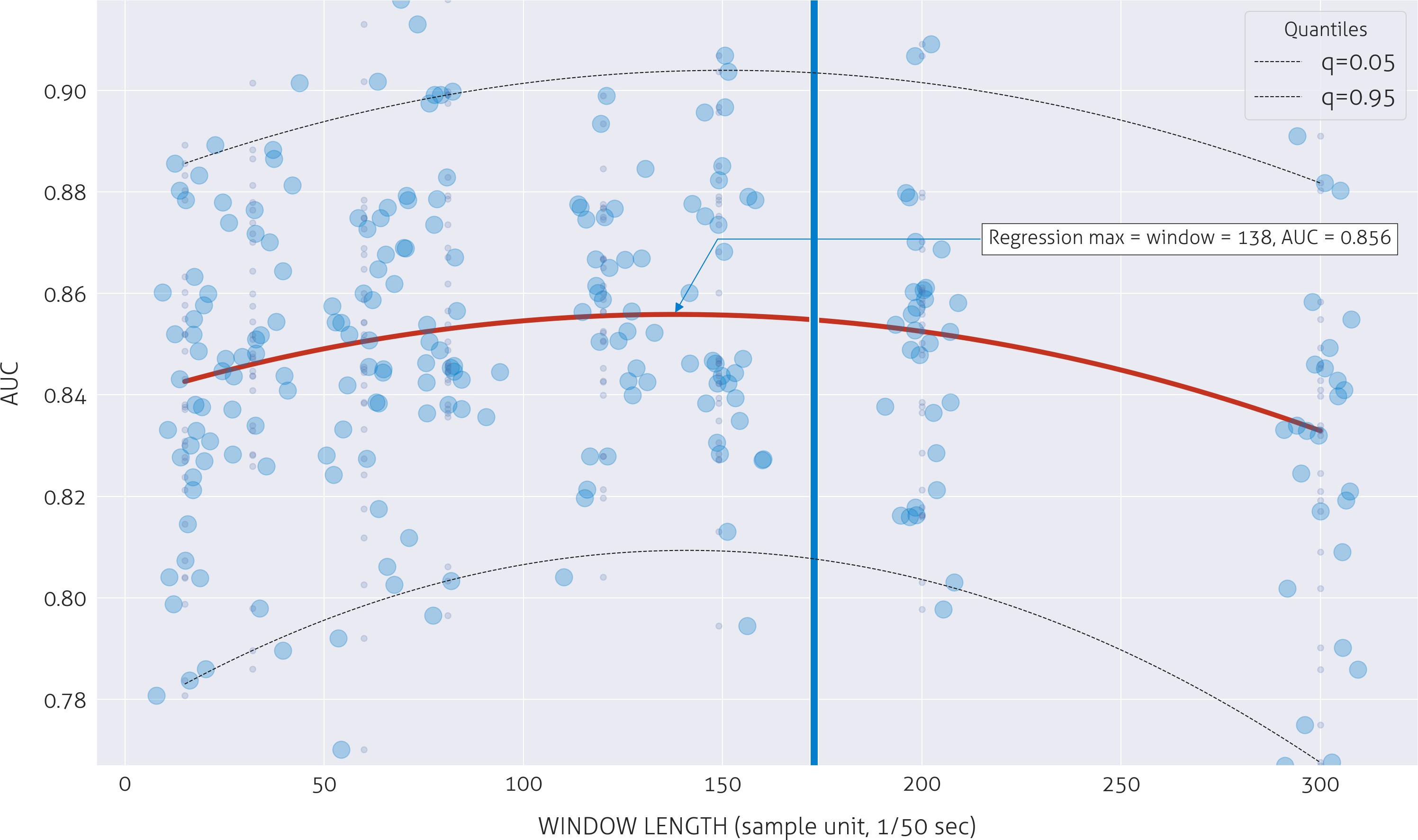


582

# A10 Fig. Performance of the machine learning classification model by 583

**window length in case of ”Stand”.** A 2-order polynomial curve fitted to the AUC 584

values of ”Stand” by window sizes. The blue vertical line shows the bout length median 585

(65), R2=0.131, p=0.000. The data points contain a horizontal jitter for better visuality. 586


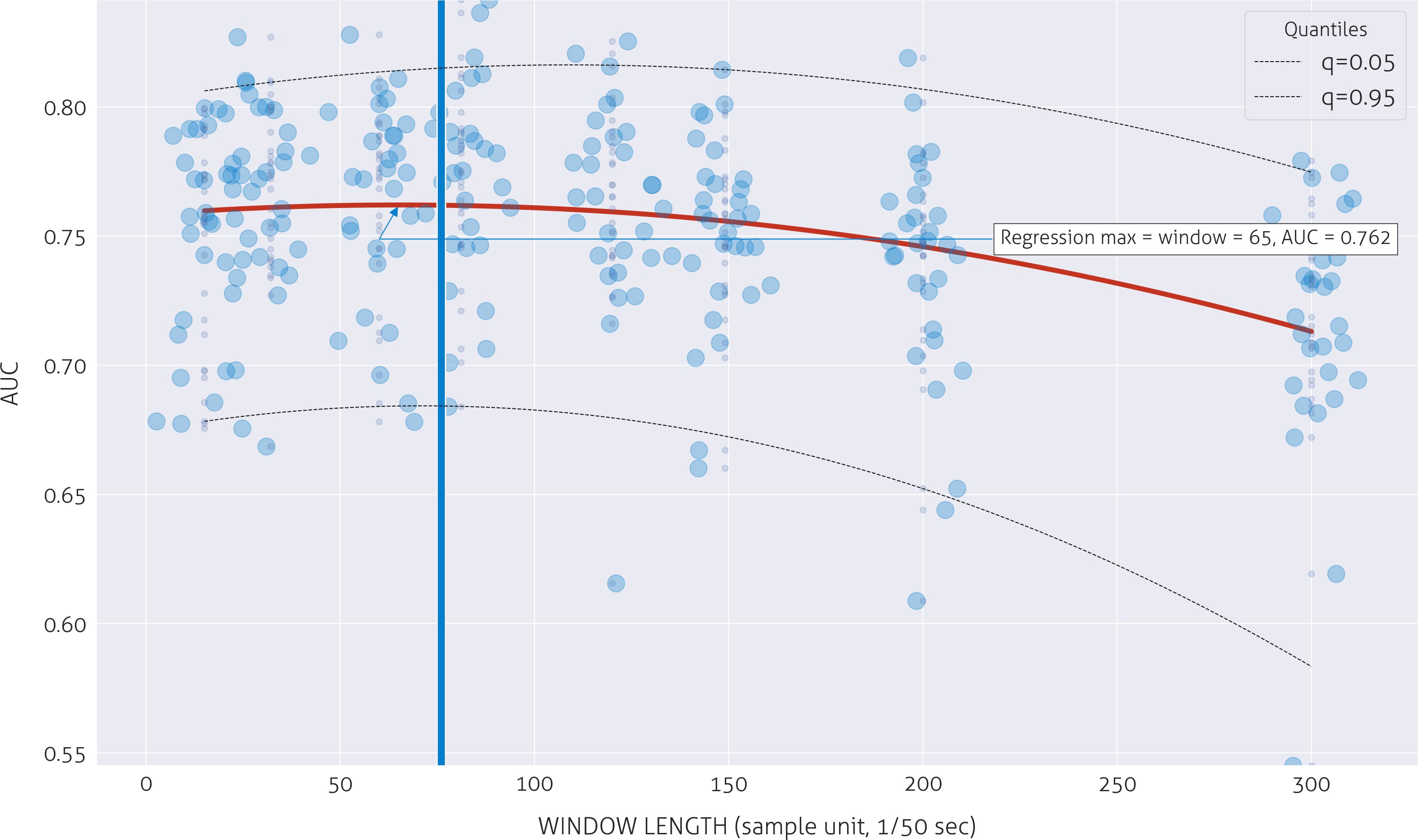


587

# A11 Fig. Performance of the machine learning classification model by 588

**window length in case of ”Walk”.** A 2-order polynomial curve fitted to the AUC 589

values of ”Walk” by window sizes. The blue vertical line shows the bout length median 590

(83) , R2=0.090, p=0.000. The data points contain a horizontal jitter for better 591

visuality. 592


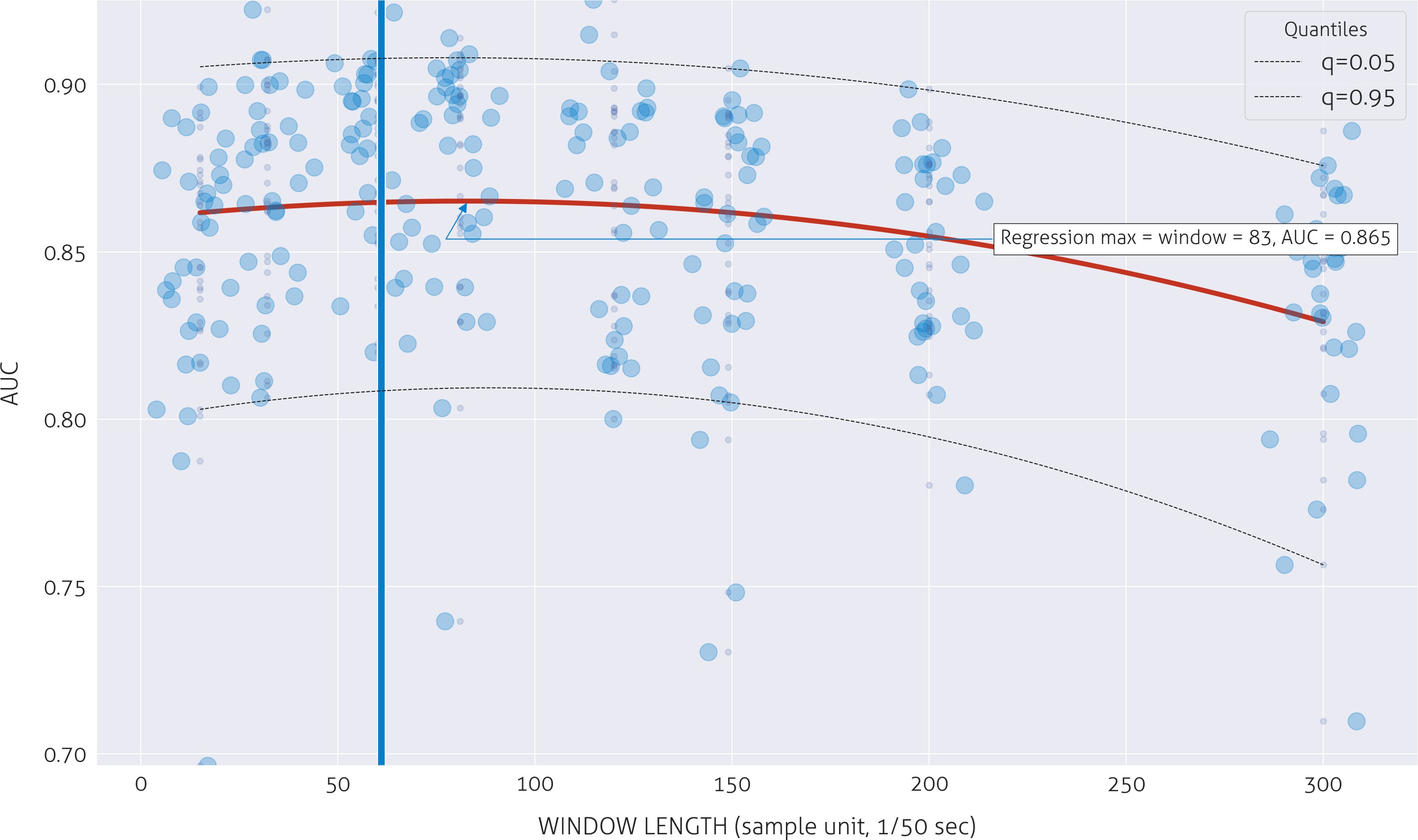


593

# A12 Fig. Performance of the machine learning classification model by 594

**window length in case of ”Trot”.** A 2-order polynomial curve fitted to the AUC 595

values of ”Trot” by window sizes. The blue vertical line shows the bout length median 596

(79), R2=0.242, p=0.000. The data points contain a horizontal jitter for better visuality. 597


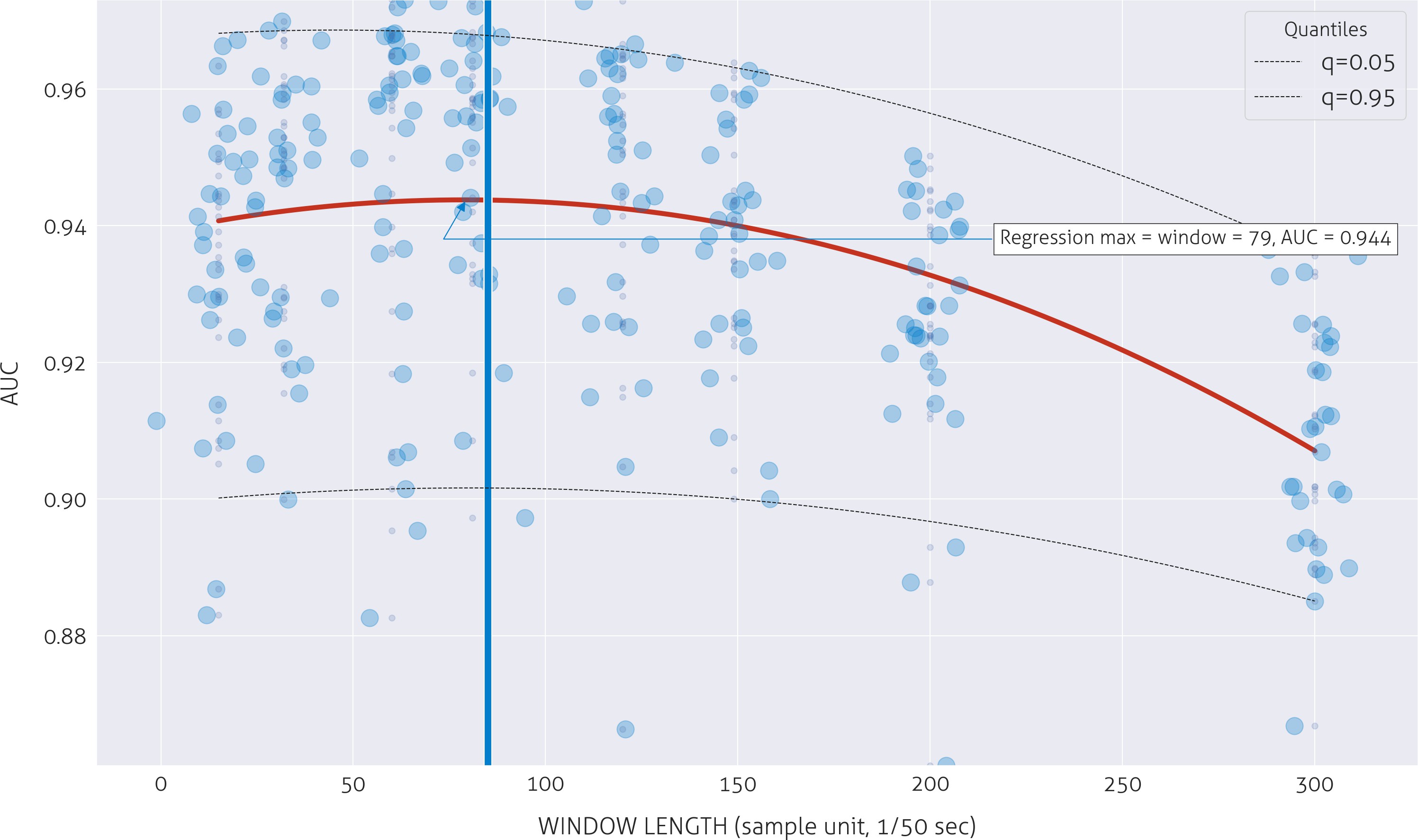


598

# A13 Fig. Performance of the machine learning classification model by 599

**window length in case of ”Run”.** A 2-order polynomial curve fitted to the AUC 600

values of ”Run” by window sizes. The blue vertical line shows the bout length median 601

(88), R2=0.323, p=0.000. The data points contain a horizontal jitter for better visuality. 602


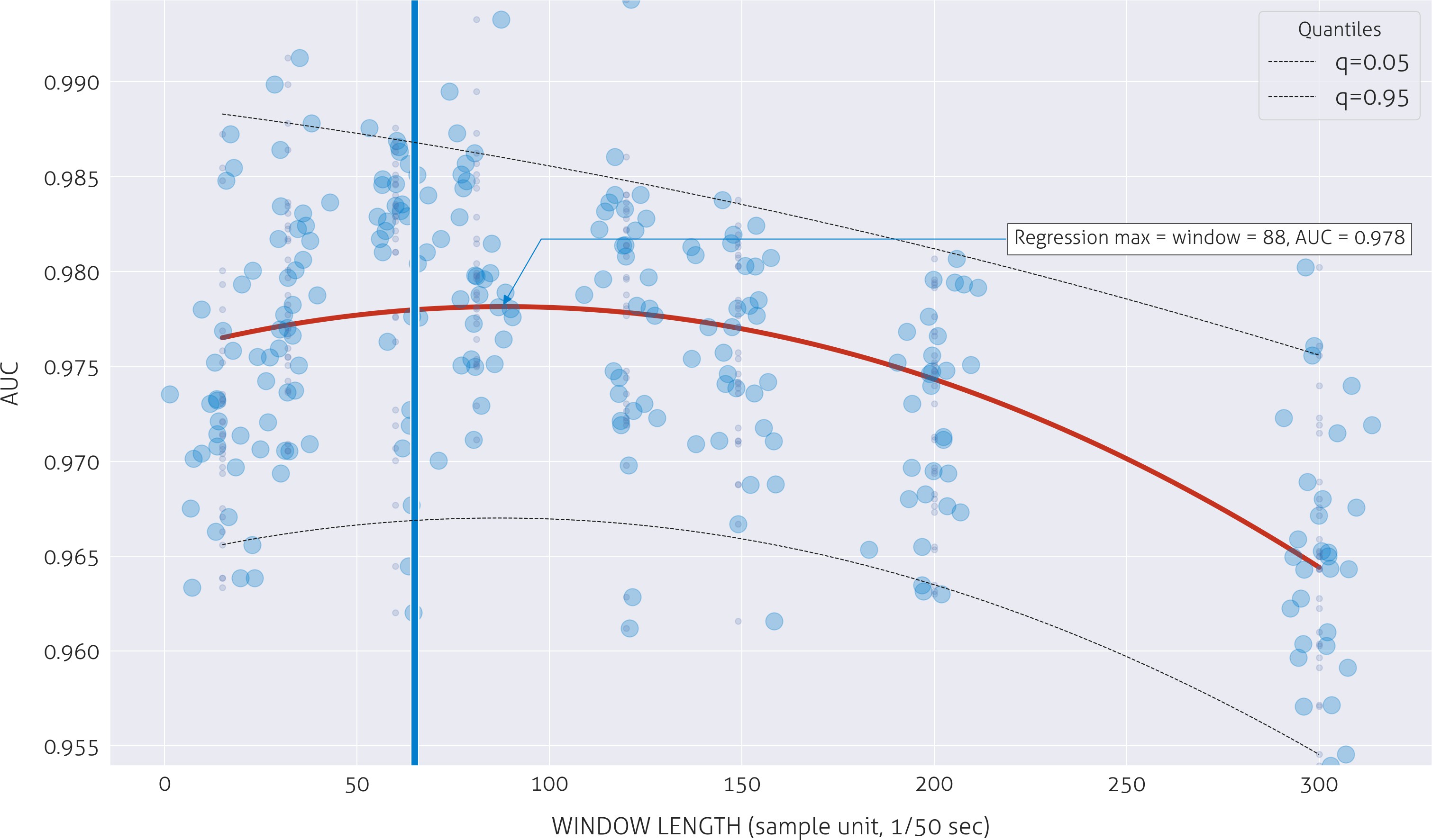


603

# A14 Fig. Performance of the machine learning classification model by 604

**window length in case of ”Eat”.** A 2-order polynomial curve fitted to the AUC 605

values of ”Eat” by window sizes. The blue vertical line shows the bout length median 606

(300), R2=0.152, p=0.000. The data points contain a horizontal jitter for better 607

visuality. 608


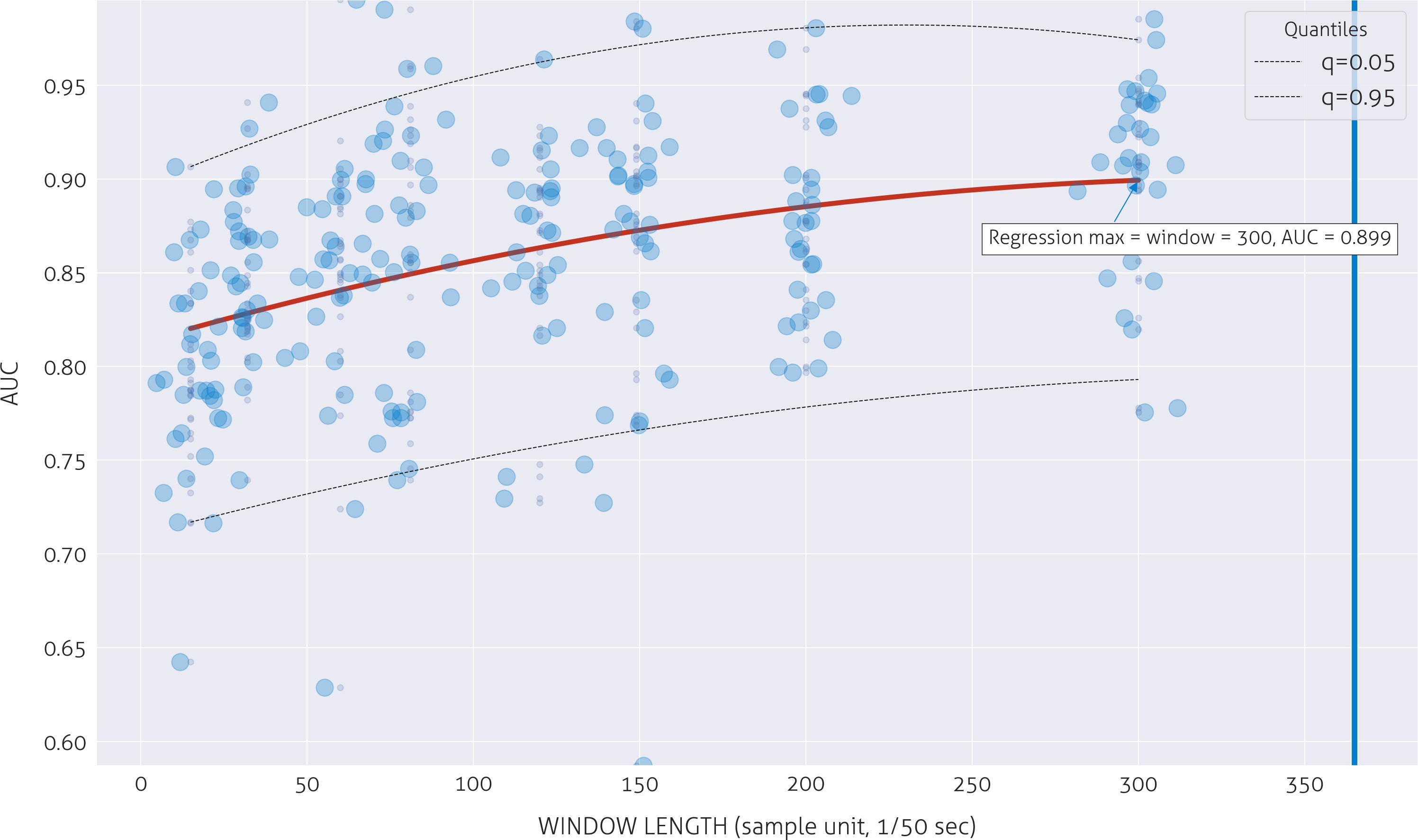


609

#
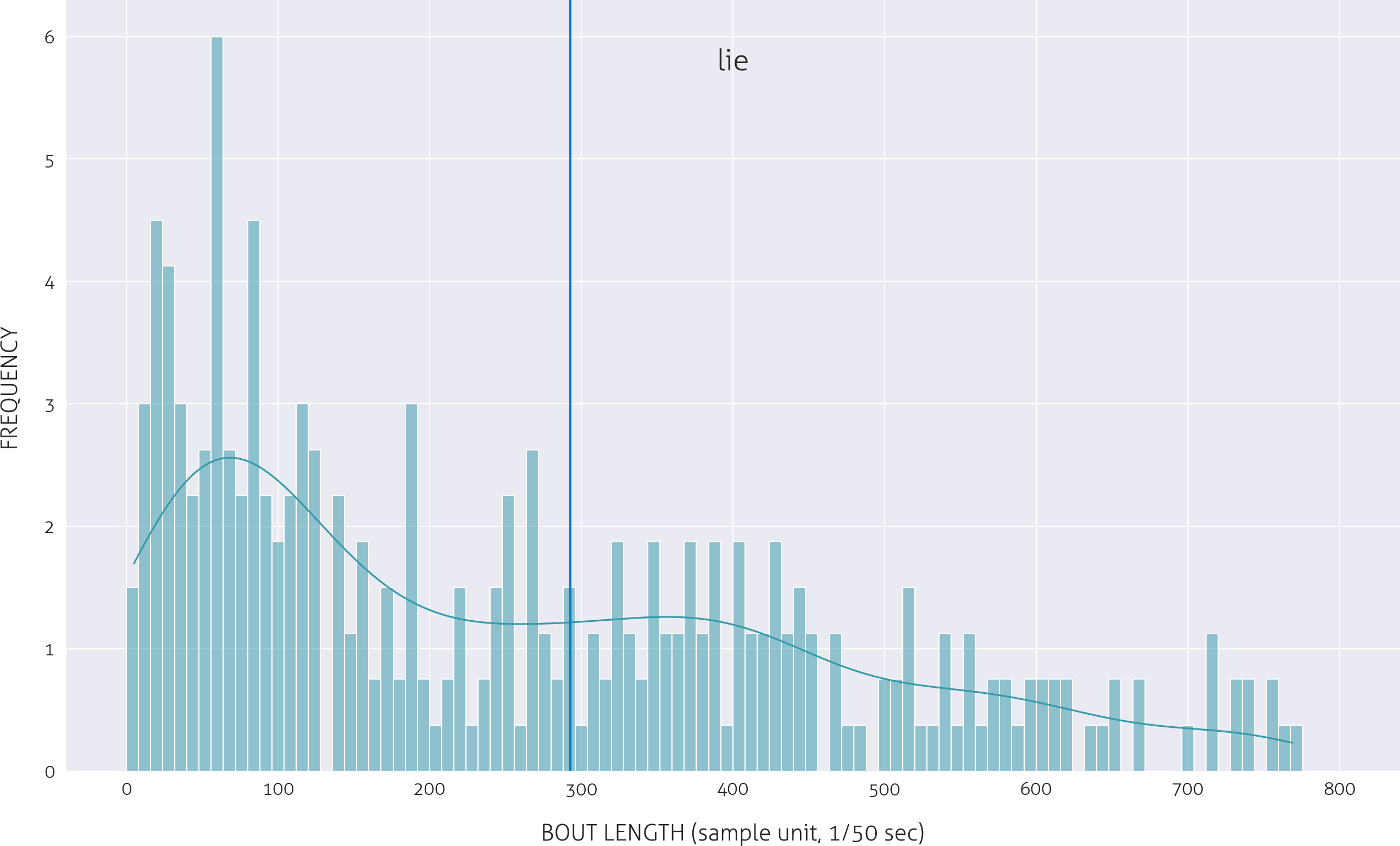
A15 Fig. Frequency distribution of bout length of ”Lie”. 610

611

#
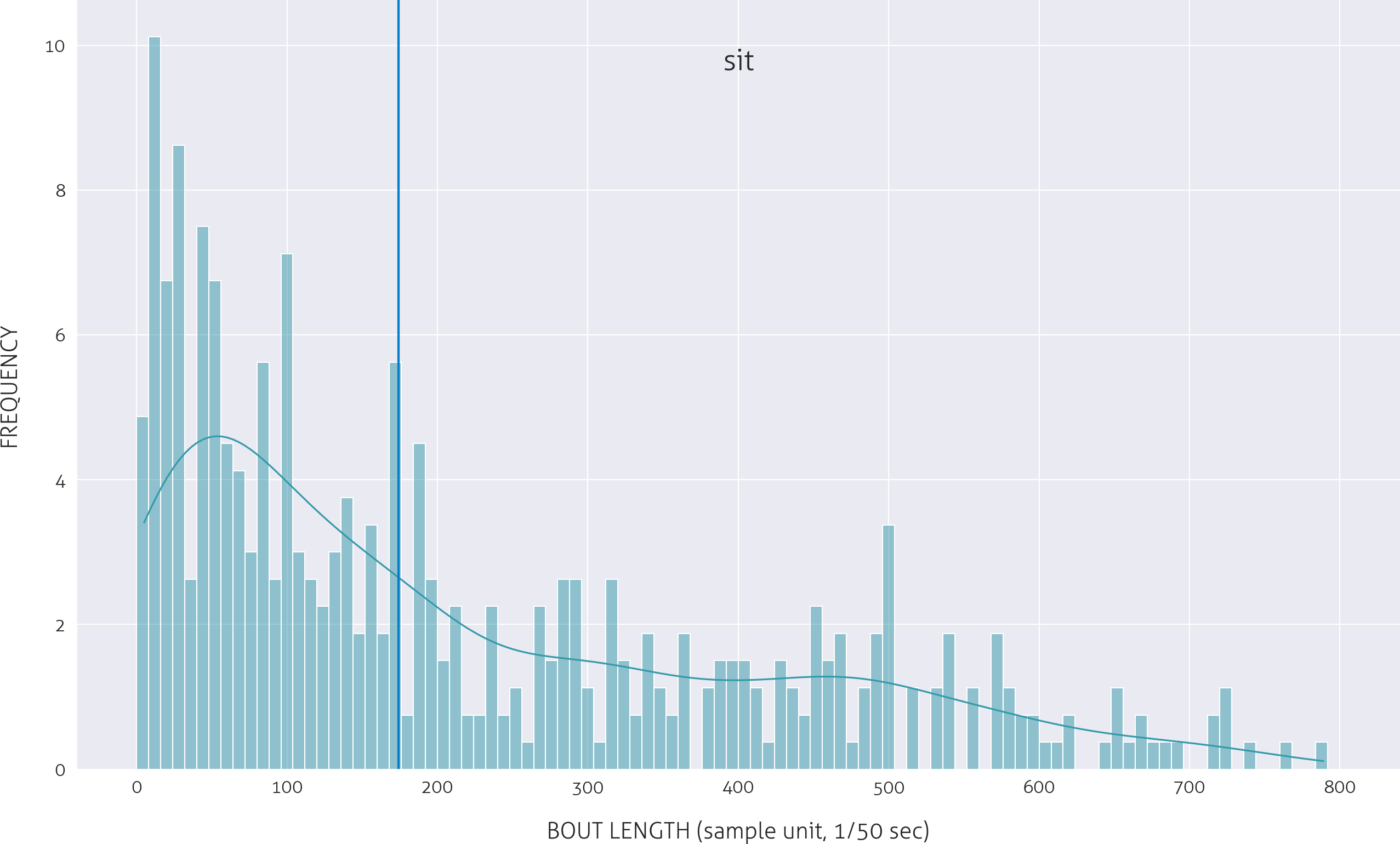
A16 Fig. Frequency distribution of bout length of ”Sit”. 612

613

#
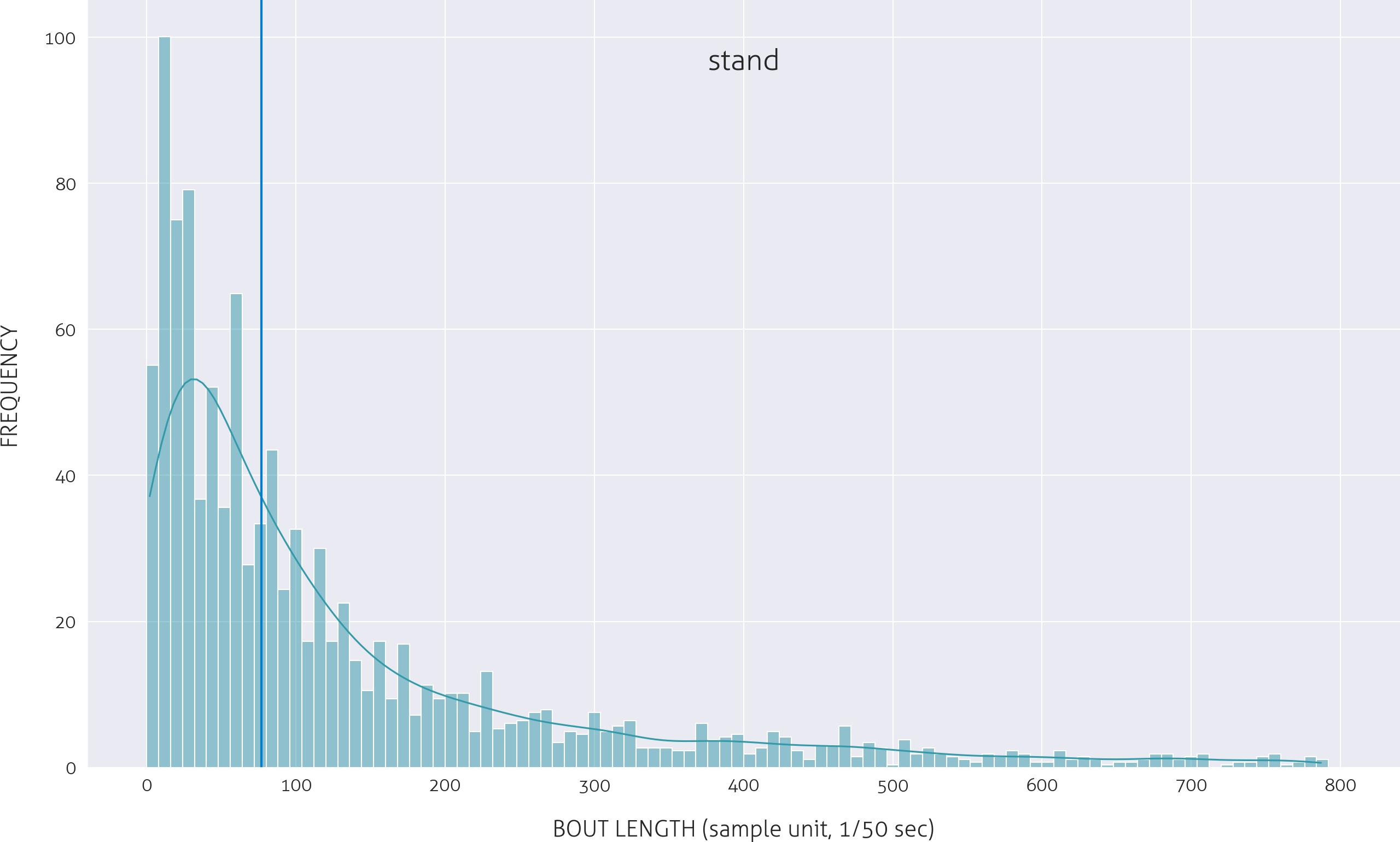
A17 Fig. Frequency distribution of bout length of ”Stand”. 614

615

#
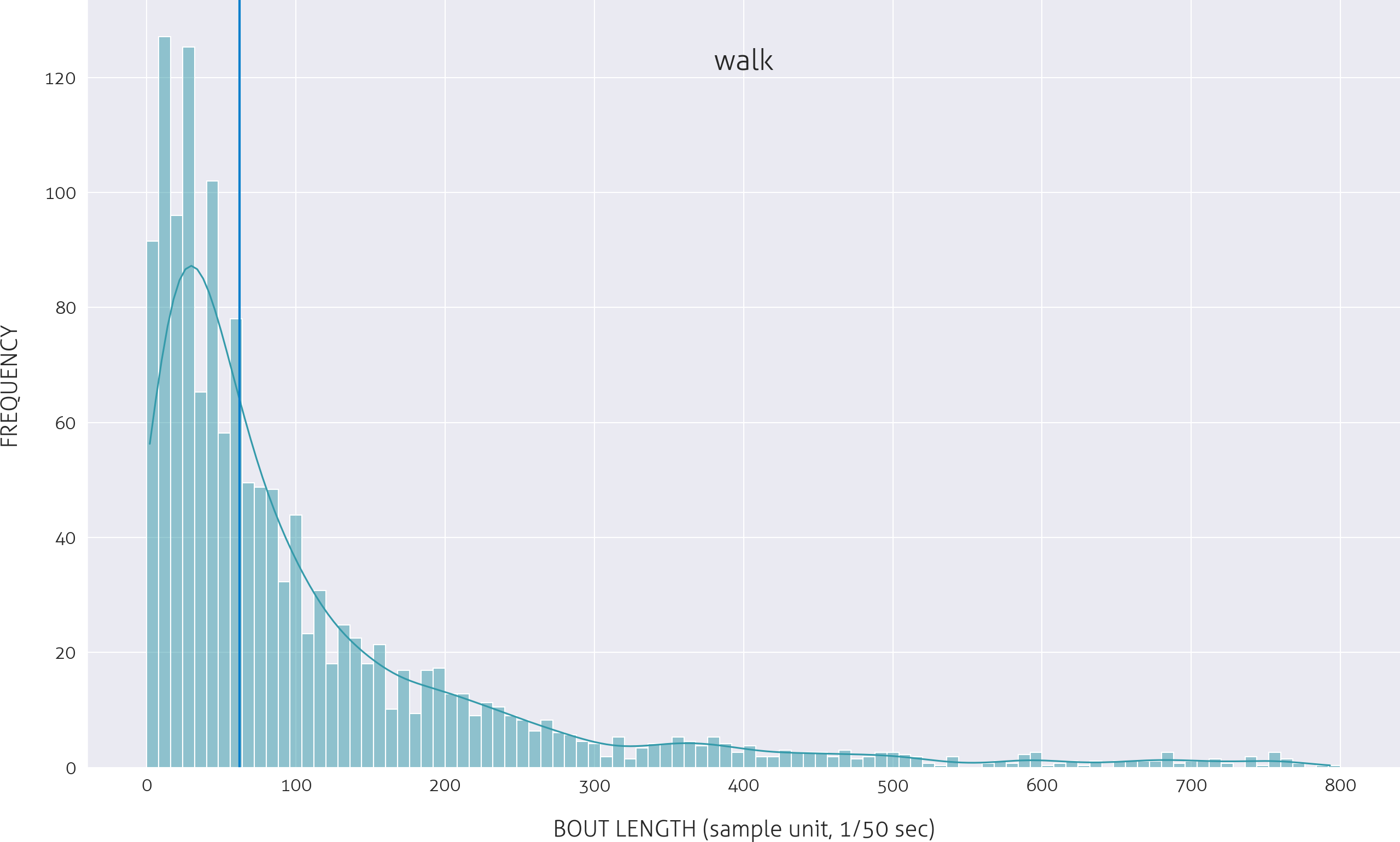
A18 Fig. Frequency distribution of bout length of ”Walk”. 616

617

#
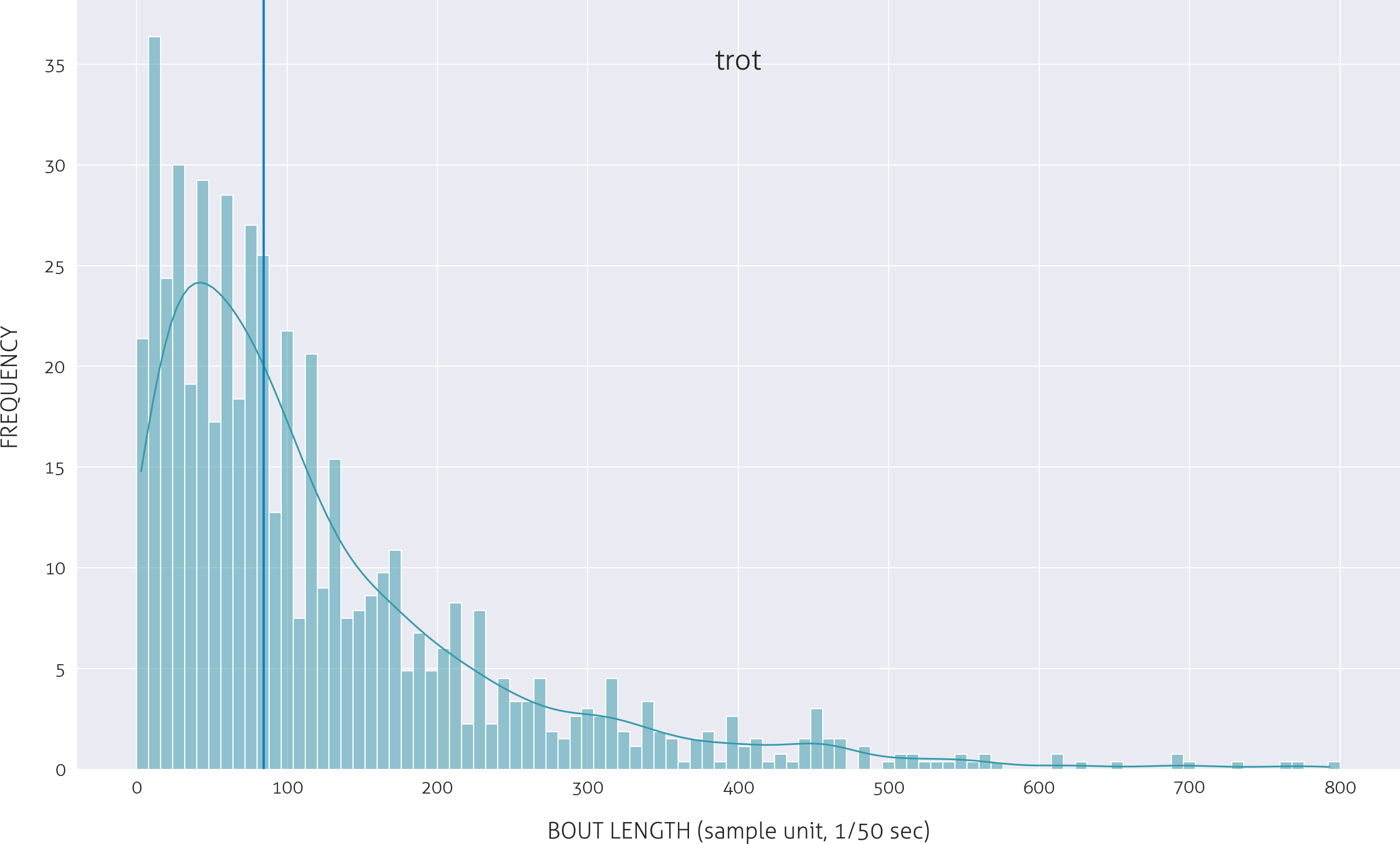
A19 Fig. Frequency distribution of bout length of ”Trot”. 618

619

#
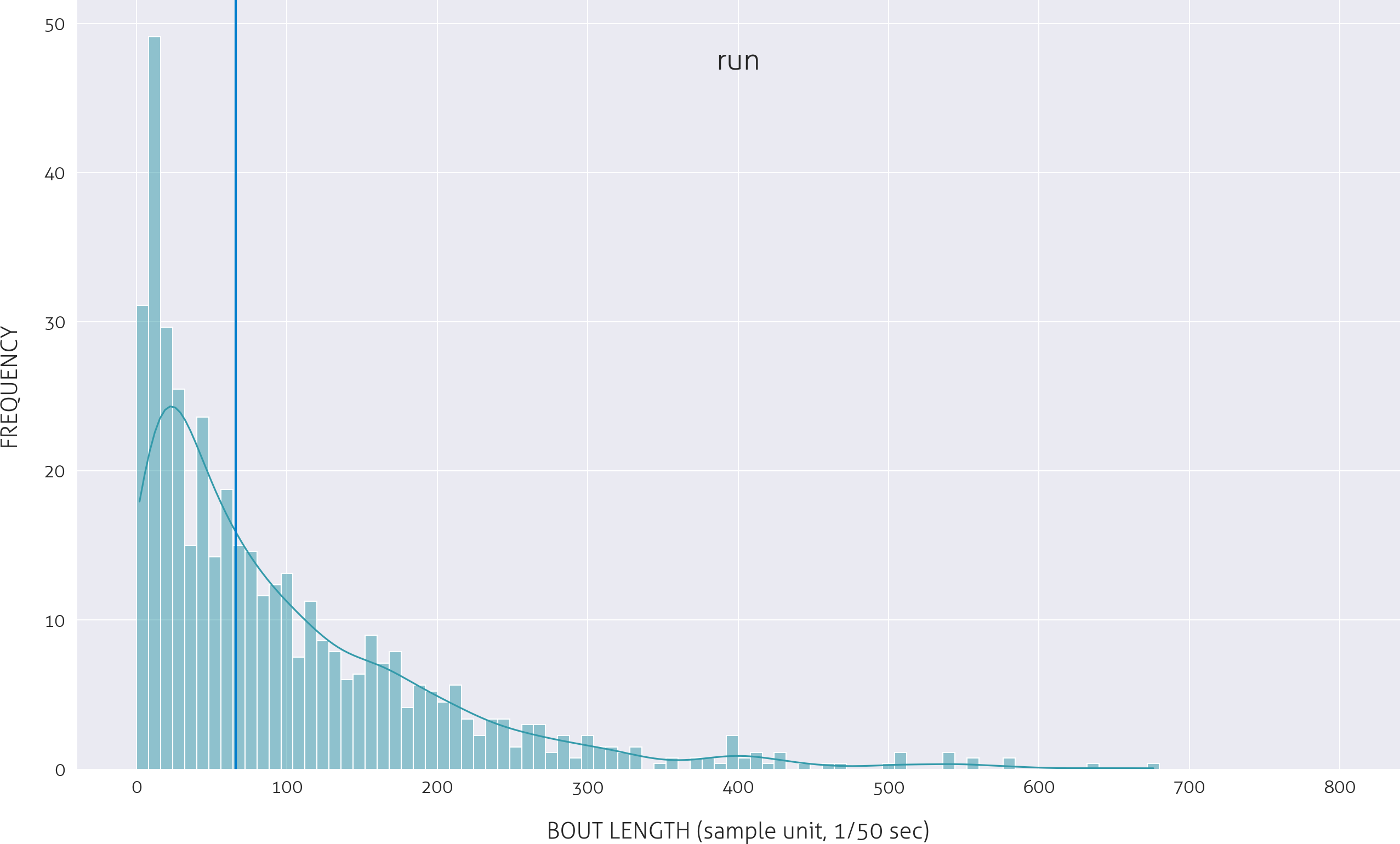
A20 Fig. Frequency distribution of bout length of ”Run”. 620

621

#
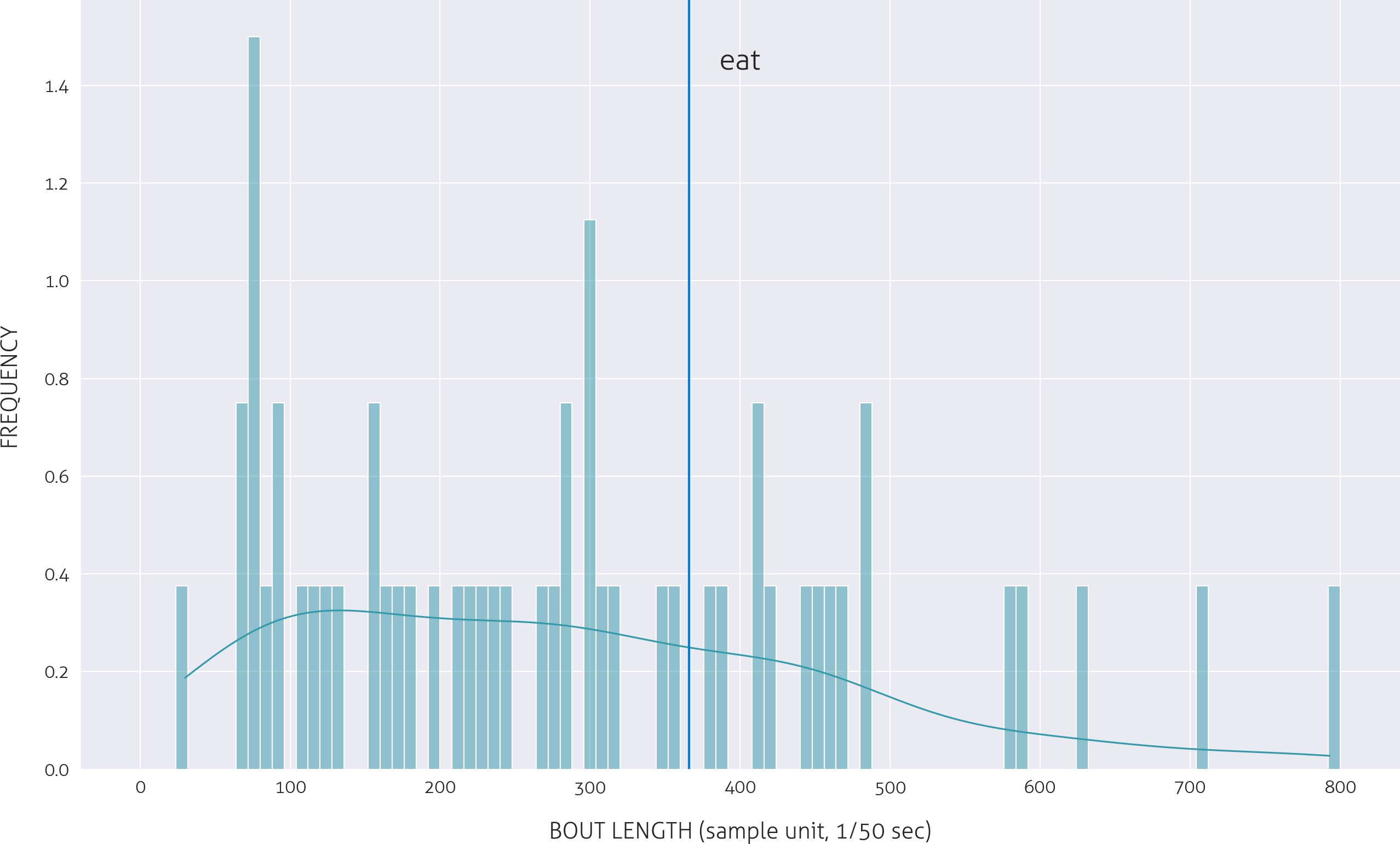
A21 Fig. Frequency distribution of bout length of ”Eat”. 622
